# Incidence and risk factors of vascular complications in people with impaired fasting glucose: a national cohort study in Korea

**DOI:** 10.1038/s41598-020-76661-7

**Published:** 2020-11-11

**Authors:** Eun Sun Yu, Kwan Hong, Byung Chul Chun

**Affiliations:** 1 National Health Insurance Service, Wonju-si, South Korea; 2 Korea University Graduate School of Public Health, Seoul, South Korea; 3 Department of Preventive Medicine, Korea University College of Medicine, Seoul, 02841 South Korea

**Keywords:** Diseases, Endocrinology, Risk factors

## Abstract

This study aimed to evaluate the risk of vascular complications of impaired fasting glucose (IFG). This population-based study included 425,608 participants from the National Health Screening Cohort in Korea in 2003 and 2004 who were followed-up until 2015. The participants were classified into normal, IFG, and diabetes groups based on fasting plasma glucose levels. Incidence rate (per 1000 person-year) was evaluated for the following vascular complications: cardiovascular (ischemic heart disease, cerebrovascular disease, arterial and capillary disease), renal, and retinal diseases. Hazard ratios (HR) of IFG for diabetes were estimated after adjusting for patient characteristics. Among the 88,330 IFG participants, the incidence of cardiovascular, chronic renal and retinal diseases were 11.52, 0.47, and 1.08 per 1000 person-years, respectively. Furthermore, IFG patients with a family history of diabetes, past history of hypertension, and high body mass index had significantly increased risk of vascular complications [adjusted HR, cardiovascular: 1.39 (95% CI 1.33–1.46); renal: 2.17 (95% CI 1.66–2.83); and retinal: 1.14 (95% CI 0.98–1.32)]. IFG patients have a substantial risk of cardiovascular, chronic renal and retinal diseases. Therefore, early preventative interventions are beneficial, especially for those with high-risk factors, in whom should emphasize on maintaining a healthy lifestyle, early screening and continuous follow-up.

## Introduction

Incidence and prevalence of diabetes and pre-diabetes are rapidly increasing among developed countries. In 2017, it was estimated that 451 million (age 18–99 years) and 374 million people worldwide had diabetes and pre-diabetes, respectively, and the estimates for diabetes were expected to increase to 693 million by 2045^1^. Impaired fasting glucose (IFG) has been considered a prediabetic state, which is associated with a relatively high risk of developing diabetes^2^. To efficiently manage this ever-growing diabetic population, early detection of prediabetes and individualized management is necessary. IFG is an important warning sign of diabetes and diabetes-related complications^3^. IFG is not only a metabolic disorder, but may also lead to cardiovascular diseases (CVD) through the damage of blood vessels by the persistently high blood glucose levels^4^. Furthermore, complications such as coronary heart disease, cerebrovascular disease, and peripheral arterial disease have been the leading cause of morbidity and mortality in diabetes^5^. IFG should not be viewed as a clinical entity in its own right rather than as a risk factor for diabetes and CVD^6^. The persistently high blood glucose levels can lead to cardiovascular diseases, diabetic nephropathy, retinopathy, and neuropathy, as well as increased death rates^7,8^.

Population-based studies from different regions of the world on the complications of diabetes among adults have been conducted^9,10^. While complications related to diabetes has been well-studied, little is known about the risk profile of IFG patients for cardiovascular, chronic renal, and retinal diseases. Moreover, the high-risk population for vascular complications among IFG patients is still unknown.

Microvascular and macrovascular complications are typical diabetic complications. It is expected that management of IFG would be more effective if the associated risks of vascular complications were elucidated. Therefore, this study aimed to compare the microvascular and macrovascular complication profiles between individuals with IFG, diagnosed diabetes, and normal glycemic status, and to define the high-risk group among IFG patients.

## Methods

### Study population and their fasting plasma glucose level

The Korean National Health Insurance Service-Health Screening Cohort (NHIS-HealS) was used. The NHIS-HealS included 514,795 participants (aged 40–79 years) randomly selected from 10% of the overall participants of the National Health Screening Program (NHSP) conducted in 2002 and 2003, who were followed-up until December 31, 2015. The NHSP cohort database contains hospital admission and outpatient visit records, medical diagnosis, death records, demographic information, drug prescriptions, and clinical information such as health questionnaire surveys, physical examination results, and biochemical results.

From the NHIS-HealS cohort, we selected 425,608 individuals with fasting glucose test results in 2003–2004. Fasting plasma glucose (FBG) was analyzed through venous blood samples after no caloric intake for at least 8 h. Based on the modified fasting plasma glucose criteria by the American Diabetes Association (ADA) in 2019^2^, participants were classified into three groups:

Normal fasting glucose (NFG, n = 273,668): FBG < 100 mg/dL and no previous diagnosis of diabetes.

Impaired fasting glucose (IFG, n = 88,330): FBG 100–125 mg/dL and no previous diagnosis of diabetes.

Diabetes mellitus (DM, n = 63,610): FBG ≥ 126 mg/dL or a past history of DM.

Considering the long follow-up period (12 years maximum), the latest data of each participant was evaluated and used for sensitivity analyses. The most recent records of the various vascular complications represented our study outcomes. Additionally, based on the Standards of Medical Care in Diabetes-2019, the IFG group was further classified into the high- and low-risk subgroups. Participants with more than one of the following risk factors were considered high-risk: family history of DM, past history of hypertension, or body mass index (BMI) > 23 kg/m^2^ (Asian).

**Figure 1:**
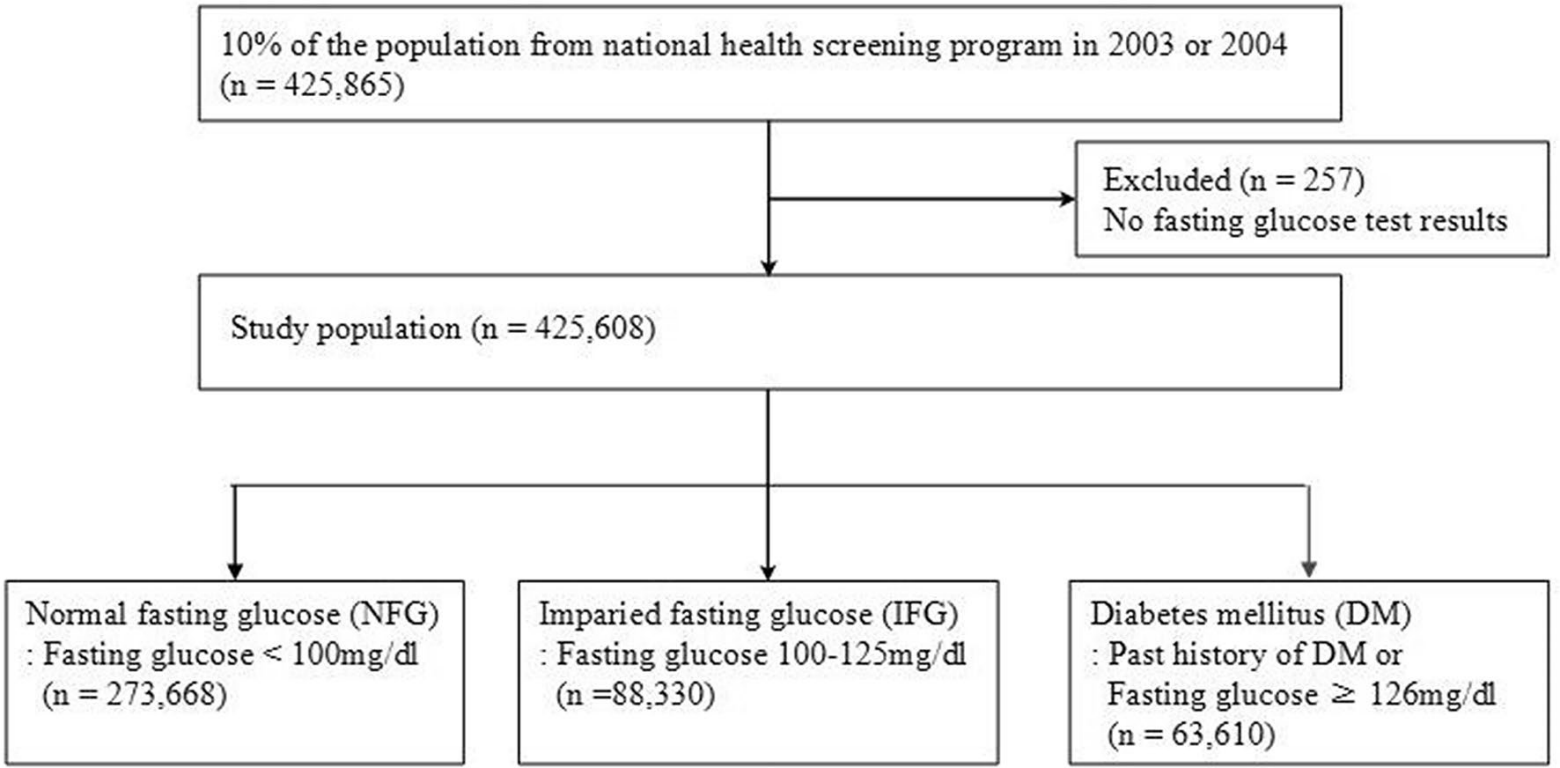
Defined study population in the health screening cohort by the National Health Insurance Service in Korea.

Past history of DM was identified either based on the 10th edition of the International Classification of Diseases (ICD-10 codes E10–E14) between 2002 and 2003, or through self-administered questionnaire. The study flowchart is presented in Fig. 1.

### Definitions of vascular complications

Two or more hospitalization days and death records based on ICD-10 codes were used as the diagnosis of complications. The follow-up period ended when the participant was diagnosed with vascular complications, died, or when the study ended (December 31, 2015). Diabetic vascular complications were classified into macrovascular and microvascular complications (Supplementary Table 1). Vascular complications were also classified into three types: cardiovascular diseases, chronic renal diseases, and retinal diseases. The disease codes were as follows: ischemic heart diseases (ICD-10: I20–I25), cerebrovascular diseases (ICD-10: I60–I68), and arterial and capillary diseases (ICD-10: I70–I79); chronic renal diseases (ICD-10: N18–N19); and retinal diseases (ICD-10: H35–H36). Retinal diseases have many reasons, including congenital diseases. Therefore, exclusion criteria were set for excluding retinal diseases by other reasons (H35.1: immature retinopathy, H35.5: hereditary retinitis).

### Statistical analysis

Patient characteristics were presented as mean ± SD (standard deviation) for continuous variables and as frequency for categorical variables. The incidence rates (cases per 1000 person-years) were calculated for the NFG, IFG, and DM groups. In addition to the crude incidence rate, adjusted hazard ratio (HR) was estimated using the Cox proportional-hazards model (reference = NFG group). Adjusted variables were selected stepwise with p-value < 0.05. The final adjusted variables included age, sex, BMI, alanine aminotransferase (ALT), total cholesterol, systolic blood pressure (BP), past history of hypertension, family history of DM, smoking status, and drinking habits. For subgroup analyses, adjusted HR was estimated in terms of the low- and high-risk groups. Rate and ratios were presented with 95% confidence intervals. p-value of < 0.05 were considered statistical significance. The proportionality and linearity of the Cox proportional-hazards model were tested with the Schoenfeld residuals and the negative-log hazard plots. All statistical analyses were performed using SAS Enterprise 7.1 (NHIS remote connection).

### Ethical approval

This project was approved by the Institutional Review Board of the Korea University (IRB: KUIRB-2019-349-1). Informed consent was waived as de-identified data were used throughout the analysis. All methods were carried out in accordance with relevant guidelines and regulations.

## Results

During the 2003–2004 period, 425,608 individuals aged 40–79 years received health screening tests. The number of participants in the NFG group was 273,668 (64.3%); IFG group was 88,330 (20.75%); and DM group was 63,610 (14.95%). During the 12-year follow-up 11,196 (4.1%) NFG and 10,664 (12.13%) IFG participants developed diabetes. In the IFG group, 11,171 (12.65%) had cardiovascular diseases, 478 (0.54%) had chronic renal diseases, and 1098 (1.24%) had retinal disease. Compared to the NFG group, IFG and DM groups were significantly associated with an increased risk of microvascular and macrovascular complications, with the DM group demonstrating the highest incidence and risk of complications in all variables, followed by the IFG group (p < 0.001). The IFG and DM groups also showed increasing trends in relative significance between the risk of vascular complications with fasting plasma glucose levels.

### General characteristics of study population

Table 1 shows the general characteristics of the study population. The proportion of males was significantly higher than females in all groups. DM participants tended to be older, exercised more, had higher systolic BP, higher BMI, as well as higher fasting plasma glucose, AST, and ALT levels (p < 0.001). Family history of DM and past history of hypertension were also significantly higher in the DM group than NFG or IFG group (p < 0.001). The IFG group contained more current smokers, consumed more alcohol, and higher total cholesterol levels as compared to the other groups.

**Table 1:** General characteristics of the study population.

Characteristics	NFG (n = 273,668)	IFG (n = 88,330)	DM (n = 63,610)	p-value
Age (years)	52.81 ± 9.28	54.12 ± 9.54	57.34 ± 9.7	< 0.01
**Sex**				< 0.01
Male	142,468 (52.06)	53,884 (61)	38,641 (60.75)	
Female	131,200 (47.94)	34,446 (39)	24.968 (39.25)	
**Smoking status**				< 0.01
None	205,297 (77.88)	63,757 (74.98)	45,965 (75.12)	
Current	58,306 (22.12)	21,275 (25.02)	15,224 (24.88)	
**Drinking frequency**				< 0.01
1–2/week	199,329 (74.13)	57,825 (66.58)	44,277 (70.87)	
3–5/week	69,556 (25.87)	29,028 (33.42)	18,202 (29.13)	
**Exercise frequency**				< 0.01
Not at all	64,404 (72.56)	21,160 (71.45)	78,533 (69.29)	
3–5/week	24,357 (27.44)	8,457 (28.55)	34,804 (30.71)	
**Past history of hypertension**				< 0.01
No	227,933 (83.29)	68,783 (77.87)	38,659 (60.78)	
Yes	45,735 (16.71)	19,547 (22.13)	24,950 (39.22)	
**Family history of DM**				< 0.01
No	235,324 (94.69)	74,791 (93.95)	51,182 (88.31)	
Yes	13,199 (5.31)	4,819 (6.05)	6,776 (11.69)	
Body mass index (kg/m^2^)	23.72 ± 2.90	24.30 ± 2.98	24.57 ± 3.13	< 0.01
Systolic BP (mmHg)	125.24 ± 17.56	129.71 ± 17.98	132.31 ± 18.61	< 0.01
Diastolic BP (mmHg)	78.59 ± 11.51	81.0 ± 11.54	81.53 ± 11.60	< 0.01
Fasting plasma glucose (mg/dL)	85.71 ± 8.42	107.99 ± 6.53	137.92 ± 64.49	< 0.01
Total cholesterol (mg/dL)	197.65 ± 36.40	204.11 ± 38.29	202.92 ± 41.33	< 0.01
Hemoglobin (g/dL)	13.84 ± 1.52	14.06 ± 1.49	14.03 ± 1.49	< 0.01
AST (U/L)	25.70 ± 15.19	27.75 ± 18.83	28.81 ± 22.51	< 0.01
ALT (U/L)	23.98 ± 18.94	27.27 ± 21.43	29.32 ± 23.55	< 0.01
Gamma GTP (U/L)	32.88 ± 43.22	43.58 ± 63.42	49.18 ± 74.49	< 0.01

Values are expressed as n (percentage) or mean ± SD (standard deviation).

*NFG *normal fasting glucose, *IFG *impaired fasting glucose, *DM *diabetes mellitus, *BP *blood pressure, *AST *aspartate aminotransferase, *ALT *Alanine aminotransferase.

### Incidence and risk of vascular complications

Incidence and risk of vascular complications of the 3 groups are presented in Table 2. The DM group was significantly associated with the highest risk of vascular complications, followed by the IFG group, and then the NFG group (p < 0.001).

**Table 2:** Incidence rate and risk of vascular complications.

	NFG (n = 273,668)	IFG (n = 88,330)	DM (n = 63.610)
**Cardiovascular diseases**			
N (%)	29,506 (10.78)	11,171 (12.65)	13,395 (21.06)
Incidence rate	9.63 (9.54–9.73)	11.52 (11.34–11.71)	20.61 (20.32–20.91)
Adjusted hazard ratio	Ref	1.03 (1.00–1.05)	1.40 (1.37–1.43)
**Ischemic heart diseases**			
N (%)	14,749 (5.39)	5,553 (6.29)	6,922 (10.88)
Incidence rate	4.71 (4.66–4.76)	5.58 (5.48–5.68)	10.18 (10.02–10.34)
Adjusted hazard ratio	Ref	1.02 (0.99–1.06)	1.43 (1.38–1.48)
**Cerebrovascular diseases**			
N (%)	15,492 (5.66)	5,942 (6.73)	7,174 (11.28)
Incidence rate	4.94 (4.89 – 4.99)	5.96 (5.86–6.06)	10.49 (10.33–10.66)
Adjusted hazard ratio	Ref	1.03 (1.00–1.06)	1.36 (1.32–1.40)
**Arterial and capillary diseases**			
N (%)	2263 (0.83)	884 (1.00)	1,238 (1.95)
Incidence rate	0.71 (0.70–0.72)	0.87 (0.85–0.89)	1.74 (1.71–1.78)
Adjusted hazard ratio	Ref	1.02 (0.94–1.11)	1.65 (1.52–1.78)
**Chronic renal diseases**			
N (%)	1114 (0.41)	478 (0.54)	1,501 (2.36)
Incidence rate	0.35 (0.34–0.35)	0.47 (0.46–0.48)	2.11 (2.08–2.14)
Adjusted hazard ratio	Ref	1.12 (1.0–1.26)	3.45 (3.16–3.76)
**Retinal diseases**			
N (%)	2425 (0.89)	840 (0.95)	1,885 (2.96)
Incidence rate	0.08 (0.07–0.08)	0.08 (0.08–0.08)	0.27 (0.26–0.27)
Adjusted hazard ratio	Ref	1.02 (0.95–1.12)	2.71 (2.53–2.90)

*NFG *normal fasting glucose, *IFG *Impaired fasting glucose. Incidence rate in cases per 1000 person-years. Adjusted for age, sex, BMI, ALT, total cholesterol, systolic BP, past history of smoking, drinking, hypertension and family history of DM.

Compared to the NFG group (9.63 per 1000 person-years), the IFG and DM groups had higher incidence and risk of cardiovascular diseases (11.52 vs 20.61 per 1000 person-year; adjusted HR 1.03 vs 1.40; and 95% CI 1.00–1.05 vs 1.37–1.43, respectively), in particular, ischemic heart diseases (5.58 vs 10.18 per 1000 person-years, respectively) and cerebrovascular diseases (5.96 vs 10.49 per 1000 person-years, respectively).

Similar to those observed in cardiovascular diseases, the risk of chronic kidney diseases increased as fasting plasma glucose levels increased. Although the absolute incidence rate of chronic renal diseases was generally lower (NFG: 0.35; IFG: 0.47; and DM: 2.11 per 1000 person-year), the IFG group had higher risk of chronic renal diseases (adjusted HR 1.12, 95% CI 1.00–1.26) than CVD. Furthermore, the DM group showed the strongest association with chronic renal diseases (adjusted HR 2.45, 95% CI 3.16–3.76).

The risk of retinal diseases was not significantly different between the NFG and IFG groups (adjusted HR 1.02, 95% CI 0.95–1.12), but was higher in the DM group (adjusted HR 2.71, 95% CI 2.53–2.90).

### Incidence and risk of vascular complications in the IFG subgroup

Table 3 shows the subgroup analysis results of the risk of vascular complications according to the level of risk. Among the 88,330 IFG participants, 64,775 were high-risk while 23,555 were low-risk. The high-risk group demonstrated higher incidence and risk of cardiovascular diseases than the low-risk group (12.57 vs 8.67 per 1000 person-years, respectively; adjusted HR 1.39; 95% CI 1.33–1.46).

**Table 3:** Comparison of risk of vascular complications between the high-risk and low-risk subgroups of the IFG group.

	Low risk (n = 23,555)	High risk (n = 64,775)
**Cardiovascular diseases total**		
N (%)	2260 (9.59)	8911 (13.76)
Incidence rate	8.67 (8.37–8.98)	12.57 (12.35–12.80)
Adjusted hazard ratio	Ref	1.39 (1.33–1.46)
**Ischemic heart diseases**		
N (%)	956 (4.06)	4597 (7.10)
Incidence rate	3.59 (3.44–3.75)	6.31 (6.18–6.43)
Adjusted hazard ratio	Ref	1.26 (1.15–1.38)
**Cerebrovascular diseases**		
N (%)	1319 (5.60)	4623 (7.14)
Incidence rate	4.97 (4.79 – 5.16)	6.32 (6.19 – 6.44)
Adjusted hazard ratio	Ref	1.19 (1.12 – 1.27)
**Arterial and capillary diseases**		
N (%)	210 (0.89)	674 (1.04)
Incidence rate	0.78 (0.74–0.81)	0.90 (0.88–0.92)
Adjusted hazard ratio	Ref	1.17 (1.00–1.38)
**Chronic renal diseases**		
N (%)	69 (0.29)	409 (0.63)
Incidence rate	0.26 (0.24–0.27)	0.54 (0.53–0.56)
Adjusted hazard ratio	Ref	2.17 (1.66–2.83)
**Retinal diseases**		
N (%)	200 (0.85)	640 (0.99)
Incidence rate	4.90 (4.62–5.20)	5.73 (5.55–5.92)
Adjusted hazard ratio	Ref	1.08 (0.38–2.65)

*IFG* Impaired fasting glucose. *High-risk *participants with one or more of the following risk factors: family history of DM, past history of hypertension, BMI > 23 kg/m^2^. Incidence rate in cases per 1000 person-years. Adjusted for age, sex, ALT, total cholesterol, systolic BP, past history of smoking and drinking.

Among the high-risk participants, the highest cardiovascular disease risk was of ischemic heart diseases (adjusted HR 1.26, 95% CI 1.15–1.38), followed by cerebrovascular diseases (adjusted HR 1.19, 95% CI 1.12–1.27), and arterial and capillary diseases (adjusted HR 1.17, 95% CI 1.00–1.38).

The high-risk group also showed significantly higher risk of chronic renal diseases (HR 2.17, 95% CI 1.66–2.83) and of retinal diseases (HR 1.08, 95% CI 0.38–2.65).

## Discussion

Large and small vessel diseases are major complications of diabetes. Diabetic patients are prone to coronary heart disease, stroke, ischemic heart disease, cerebrovascular disease, peripheral vascular disease, retinopathy, and chronic kidney disease^11–14^. In a variety of ways, these conditions can be attributed to prolonged hyperglycemia.

This study included participants aged 40–79 years old with different blood glucose categories with a 12-year follow-up period, and examined the incidence and risk of vascular complications in terms of cardiovascular, chronic renal, and retinal diseases. The DM group demonstrated the highest incidence and risk of all 3 vascular complications, followed by the IFG group. To our knowledge, this is the first study that compared the risk of vascular complications using incidence rates between different fasting blood glucose groups. It was confirmed that not only DM patients, but even those with IFG, had a substantial risk of vascular complications. This study also showed that past history of hypertension, family history of DM and high BMI were high-risk factors for vascular complications in IFG patients.

Based on a previous systematic review^15^ on the relationship between pre-diabetes and CVD risk, 8 publications reported that IFG (100–125 mg/dL) showed a relative rate (RR) of 1.18 (95% CI 1.09–1.28). Another study^16^ argued that interventions for vascular diseases should be indicated if intermediate hyperglycemia actually increased the risk of CVD by 20%. Diabetic complications involving atherosclerotic diseases have been more concerning than glucose-centric small vessel diseases^17,18^, and the advancement of arteriosclerosis among prediabetic and diabetic patients have been much faster than that in normal individuals. Another study supported that this accelerated progression of arteriosclerosis often lead to coronary heart disease and stroke^19^, reporting an increased CVD risk in IFG and DM patients with adjusted HR of 1.03 (95% CI 1.09–1.28) and 1.39 (95% CI 1.33–1.46), respectively. In line with this, our cohort study confirmed that efforts to lower blood sugar levels to normal ranges can reduce the risk of CVD. Aneurysm is an important vascular disease. However, in this study, the risk factors of aneurysm remain unclear because there were no statistically significant differences.

The issue of whether intermediate hyperglycemia (pre-diabetes) can induce diabetic microvascular disease has been discussed in detail in the WHO report of 2006^20^, which concluded that increased plasma glucose level is a predicting factor for microvascular disease and mortality. Many studies have reported that small vessel disease is very much related to hyperglycemia, and the only way to prevent or delay microvascular diseases in pre-diabetic patients is to prevent the development of diabetes^21,22^. Chronic renal failure usually occurs about 15–30 years after the onset of type 1 and 2 diabetes^23^, so the incidence rate is low but the potential risk is quite high. Although the absolute incidence rate of chronic renal disease was generally lower, our data corroborated the association between IFG and the high risk of renal complications. In line with this, a relatively high prevalence of decreased kidney function was shown in prediabetic patients of the United State^24^. A cross-sectional study demonstrated that glomerular filtration rate (GFR) is elevated among prediabetic and newly diagnosed diabetic individuals both at baseline and during at least 4 years of follow-up. These findings were consistent with early diabetic glomerular hyperfiltration^25^. Similar to our study, a population-based study in China categorized glycemia into diagnosed diabetes, undiagnosed diabetes, prediabetes, normal glycemia, and estimated the prevalence of kidney diseases. The prevalence of albuminuria, decreased kidney function and chronic kidney diseases increased with higher glycemic levels^26^.

Our findings showed that the risk of retinal disease increased in the high-risk IFG group. Funagata et al. demonstrated that retinopathy was associated with high BMI, and both impaired glucose tolerance and IFG^27^. Retinopathy may occur even before the development of type 2 diabetes, and was associated with hypertension and high BMI, which are the main features of metabolic syndrome^28,29^.

IFG with high-risk factors such as family history of diabetes, history of hypertension and high BMI had higher incidence of vascular complications, and were associated with elevated risk of cardiovascular, retinal and chronic renal diseases. Diabetes and its complications are complex and multifactorial, involving both environmental and genetic components^30^. Genetic determinants of diabetes are likely to be more common in patients with early onset diabetes^31^. Numerous genetic studies have demonstrated a clear genetic component to both diabetes and its complications^32–34^. The risk factors for individual and concurrent vascular complications include family history, hypertension, age, family history, duration of diabetes, and BMI. It is well known that obesity, in particular, predisposes to type 2 diabetes, a condition in which even moderate weight loss can improve insulin resistance and chronic hyperglycemia, both of which are related to microvascular complications^35,36^. Taken together, these data suggest that several variables significantly associate with one or more microvascular and macrovascular complications.

### Study limitations and strengths

This study had several limitations. First, fasting blood glucose may have been misclassified as only one measurement was taken at baseline. However, fasting plasma glucose was evaluated after no caloric intake for at least 8 h and false-positive cases were therefore likely to be rare. Second, a combination of 75 g oral glucose tolerance test (OGTT) and glycosylate hemoglobin (HbA1c) is required for a more accurate diagnosis of impaired fasting glucose. However, OGTT and HbA1c were not included due to a lack of NHIS-HEALS data. Third, the use of blood pressure and lipid drugs were not distinguished among these participants and the clustering of multiple complications was not considered. Despite the use of a large, nationally representative cohort with a long follow-up period which covered a substantial range of validated vascular complication cases, further studies using more accurate classification of diabetic complications and the effect of drugs are needed to validate our findings (Supplementary Table 1).

## Conclusions

This longitudinal study revealed that impaired fasting glucose increased the risk of vascular complications (cardiovascular disease, chronic renal disease, retinal disease). Early preventative interventions are beneficial, especially among IFG patients with high-risk factors such as family history of DM, past history of hypertension, and high BMI. We recommend that a healthy lifestyle, early screening, and continuous follow-up should be emphasized in individuals with impaired fasting glucose.

## Supplementary information

Supplementary Table 1.
