# Supplementary material for: Incidence and risk factors of vascular complications in people with impaired fasting glucose: a national cohort study in Korea
Source: Sci Rep. 2020 Nov 11;10:19504. doi: 10.1038/s41598-020-76661-7 (PMC7659344; doi:10.1038/s41598-020-76661-7)
Supplement: Supplementary file 1 — Supplementary Table 1. [file 41598_2020_76661_MOESM1_ESM.docx]

**Incidence and risk factors of vascular complications in people with impaired fasting glucose: A national cohort study in Korea**

Eun Sun Yu¹^,^², Kwan Hong²^,^³, and Byung Chul Chun²^,^³^*^

¹National Health Insurance Service, Korea

²Korea University Graduate School of Public Health, Seoul, Korea

³Department of Preventive Medicine, Korea University College of Medicine, Seoul, Korea

Supplementary Table 1. Definitions of vascular complications based on the 10^th^ revision of the International Statistical Classification of Disease and Related Health Problems (ICD-10).

| ICD-10 | Diagnosis | |
| --- | --- | --- |
| **Cardiovascular diseases** | | |
| Ischemic heart diseases | | |
| I20 | Angina pectoris | |
| I21 | Acute myocardial infarction | |
| I22 | Subsequent myocardial infarction | |
| I23 | Certain current complications following acute myocardial infarction | |
| I24 | Other acute ischemic heart diseases | |
| I25 | Chronic ischemic heart disease | |
| Cerebrovascular diseases | | |
| I60 | Subarachnoid hemorrhage | |
| I61 | Intracerebral hemorrhage | |
| I62 | Other nontraumatic intracranial hemorrhage | |
| I63 | Cerebral infarction | |
| I64 | Stroke, not specified as hemorrhage or infarction | |
| I65 | Occlusion and stenosis of precerebral arteries, not resulting in cerebral infarction | |
| I66 | Occlusion and stenosis of cerebral arteries, not resulting in cerebral infarction | |
| I67 | Other cerebrovascular diseases | |
| I68 | Cerebrovascular disorders in diseases classified elsewhere | |
| Arterial and capillary diseases | | |
| I70 | | Atherosclerosis |
| I71 | | Aortic aneurysm and dissection |
| I72 | | Other aneurysm and dissection |
| I73 | | Other peripheral vascular diseases |
| I74 | | Arterial embolism and thrombosis |
| I77 | | Other disorders of arteries and arterioles |
| I78 | | Diseases of capillaries |
| I79 | | Disorders of arteries, arterioles and capillaries in diseases classified elsewhere |
| **Chronic renal diseases** | | |
| N18 | | Chronic kidney disease |
| N19 | | Unspecified kidney failure |
| **Retinal diseases** | | |
| H35  H36 | | Other retinal disorders (Excludes: H35.1, H35.5)  Retinal disorders in diseases classified elsewhere |
